# Nutrition and Aging Across the Plains: A Qualitative Study

**DOI:** 10.1093/geroni/igaf122.2434

**Published:** 2025-12-31

**Authors:** Emily Frankel, Lindsay Wilkinson, Christopher Kelly

**Affiliations:** University of Nebraska Medical Center, Omaha, Nebraska, United States; University of Nebraska Omaha, Omaha, Nebraska, United States; University of Nebraska Omaha, Omaha, Nebraska, United States

## Abstract

Nutrition is a vital driver of health, playing a key role in health promotion and overall well-being across the lifespan. Older adults, who face morbidity and compromised health outcomes, can leverage dietary habits that support cognitive functioning, decrease inflammation, increase longevity, and improve quality of life. Unfortunately, communities experience heterogeneity in food availability, access, and education, such that rural communities tend to be older, sicker, and economically disadvantaged, compared to urban communities. To understand the nutritional perceptions and experiences of older adults residing in rural and urban communities in Nebraska, 24 older adults (n = 8), medical providers (n = 8), and key partners (n = 8) participated in semi-structured interviews between October 2024 and November 2024. A thematic analysis revealed four main themes: 1. Relationships and Social Connectedness, 2. Education and Empowerment, 3. Balance and Wellness, and 4. Access and Independence. Ascertaining perspectives from rural- and urban-dwelling individuals demonstrated the role of the built environment on health behaviors. Rural residents were impacted disproportionately and more likely to report a lack of or diminished choice of grocery stores or healthy food establishments. Transportation also varied greatly between rural and urban communities, where rural communities often lacked public transportation. The affordability of produce was demonstrably different in rural vs urban communities and created a barrier for older adults in rural communities to access produce as readily as their urban counterparts. These findings offer innovative and valuable insight into enhancing community programming and resource development for rural and urban communities, ultimately supporting health and well-being through nutrition-based initiatives.

